# Effective hole conductivity in nitrogen-doped CVD-graphene by singlet oxygen treatment under photoactivation conditions

**DOI:** 10.1038/s41598-022-12696-2

**Published:** 2022-05-24

**Authors:** Giuseppe Valerio Bianco, Alberto Sacchetti, Marco Grande, Antonella D’Orazio, Antonella Milella, Giovanni Bruno

**Affiliations:** 1 Institute of Nanotechnology, CNR‑NANOTEC, Dipartimento Di Chimica, Università Di Bari, via Orabona, 4, 70126 Bari, Italy; 2 Dipartimento di Chimica, Università Di Bari, via Orabona, 4, 70126 Bari, Italy; 3 Dipartimento di Ingegneria Elettrica e dell’Informazione, Politecnico Di Bari, via Orabona,4, 70123 Bari, Italy

**Keywords:** Chemistry, Materials science, Nanoscience and technology

## Abstract

Nitrogen substitutional doping in the π-basal plane of graphene has been used to modulate the material properties and in particular the transition from hole to electron conduction, thus enlarging the field of potential applications. Depending on the doping procedure, nitrogen moieties mainly include graphitic-N, combined with pyrrolic-N and pyridinic-N. However, pyridine and pyrrole configurations of nitrogen are predominantly introduced in monolayer graphene:N lattice as prepared by CVD. In this study, we investigate the possibility of employing pyridinic-nitrogen as a reactive site as well as activate a reactive center at the adjacent carbon atoms in the functionalized C–N bonds, for additional post reaction like oxidation. Furthermore, the photocatalytic activity of the graphene:N surface in the production of singlet oxygen (^1^O_2_) is fully exploited for the oxidation of the graphene basal plane with the formation of pyridine N-oxide and pyridone structures, both having zwitterion forms with a strong p-doping effect. A sheet resistance value as low as 100 Ω/□ is reported for a 3-layer stacked graphene:N film.

## Introduction

Since the publication on graphene by Geim and Novoselov in 2004, and after more than one hundred thousand publications, there is no doubt that the success of graphene lies in its fascinating properties such as one-atom thickness, transparency, thermally and electrically conductivity, high mobility of electrons and flexibility^1–6^. Nowadays, however, the good reputation of graphene as a promising material for various applications^7–11^, is also due to the possibility to modulate its chemical and physical properties through chemical functionalization and, in particular, doping^12–16^.

Graphene doping can be performed through two methodologies: (i) surface chemical functionalization, both covalent (i.e., bonding new atoms or molecules to the carbon honeycomb pattern) and non-covalent (i.e., adsorption of gas, metal and organic molecule) and (ii) substitutional doping in the hexagonal lattice of the graphene with heteroatoms, mainly nitrogen and boron, capable to control the n-type and p-type character of the graphene, respectively^14^. Specifically, when nitrogen atoms are substitutionally incorporated into the π-lattice of the carbon ring, they add resonant electrons, which provide n-doped graphene sheets. Nevertheless, the landscape of the nitrogen functional forms possibly present in nitrogen-doped graphenic materials is very rich^17–19^. In CVD nitrogen-doped graphene, depending on the doping procedure, the nitrogen moieties include graphitic-N, combined with pyrrolic and pyridinic nitrogen^18^. Among these bonding configurations, graphitic-N induces an n-type doping effect, whereas pyrrolic-N and pyridinic-N may result in either weak n-type or p-type doping^16,20^. To date, there are quite a number of studies focused on the growth of N-doped graphene by direct or post treatment processes, which show the capability of tuning graphene chemical and physical properties^21–25^. However, none of them has reported that the nitrogen functionalities inserted in the basal plane of the carbon lattice may be further modified to further improve the conductivity of the layer. Thus, from a chemical standpoint, N-doped graphene is an appealing graphene type, because nitrogenous functionalities and, in particular, pyridine nitrogen can be a reactive center as well as activate a reactive center at the adjacent carbon atoms in the functionalized C-N bonds for additional post reaction like oxidation^26,27^. In addition, the presence of these N-functionalities in the graphene structure, besides changing the carrier density (i.e., doping), gives an interesting catalytic activity of the graphene surface, such as the well investigated activated generation of reactive oxygen species (ROS)^28–31^. Among these new “catalytic” capabilities, the production of singlet oxygen (^1^O_2_) by both (i) activating the dissociation of peroxydisulfate^28^ and (ii) the photosensitize excitation of molecular oxygen^30^ is of particular interest. In fact, singlet oxygen (also known as singlet delta oxygen ^1^O_2_(^1^Δg)), being a non-radical reactive oxidizing specie, does not intervene on the conjugated double bond as it happens for radical species (oxygen atoms, OH radicals, ozone) and shows higher reactive selectivity also because of its electrophilic nature.

In this paper, we report on N-doped graphene, grown by CVD, and its subsequent chemical modification to improve the p-type conductivity, taking advantage of the presence of nitrogen functionalities as new active sites for selective reactions, without affecting the sp^2^ graphene network. As for the CVD growth of N-doped graphene on copper foil, the major challenges are how to control the insertion of the different N-functionalities. Specifically, in order to realize an increase in p-type doping, we need to minimize the graphitic nitrogen in favour of pyrrolic and, even better, pyridinic nitrogen; this is because each nitrogen substituting a graphitic carbon increases the number of conjugated *π*-electrons, thus improving the n-type conductivity. It is important to underline that N-graphitic atom, like carbon atom in pure graphene, has a low reactivity. Among the different methodologies proposed in literature, we opted for the CVD growth of N-functionalized graphene (graphene:N) via N atoms embedded into Cu substrate and used as a nitrogen solid source^24^. We show that this approach allows obtaining N-graphene layers with small grain size and therefore a high density of structural defects, such as grain boundaries and carbon vacancies, which are the preferred sites for the inclusion of pyridinic- and pyrrolic-nitrogen. From this point of view, in other words, graphene grain-boundary defects do not damage the material properties but rather improve them^32^. The post-growth treatment of N-doped graphene to realize chemical modifications at nitrogen reactive sites is performed by photoirradiation with Xe lamp of N-graphene sample in air. The chemistry, involving the interaction of singlet oxygen photo-catalytically activated on N-graphene, is highly selective towards pyridine active sites leaving the basal plane components intact. We show that the pyridine ring almost disappear in favour of pyridine N-oxide and pyridone structures, both having a strong p-doping effect.

## Results and discussion

**Figure 1:**
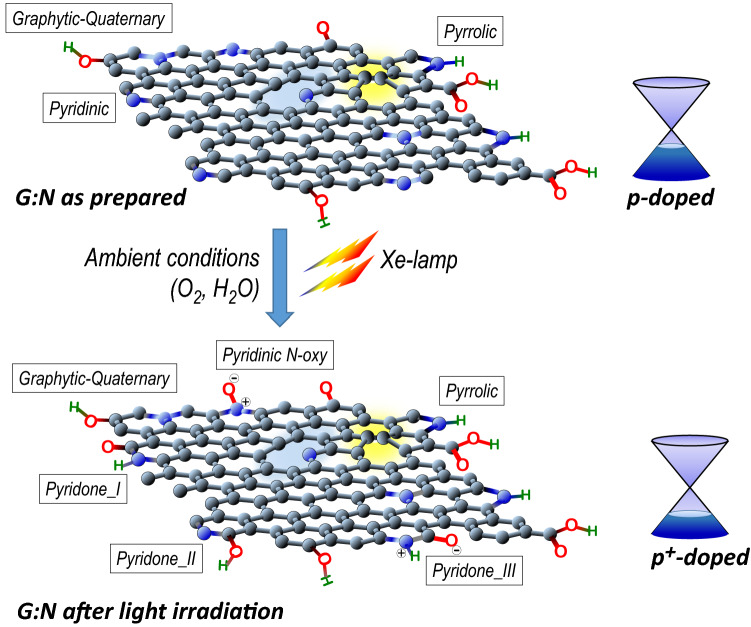
Schematic representation of the N-doped graphene as prepared and after light irradiation under ambient conditions. The blue spheres represent nitrogen atoms. The three different C–N bonding configurations inserted into the graphene network are evidenced in the as prepared G:N, as well as the typical carbon vacancy (blue area) and the Stone–Wales ( yellow area) defects. Following light irradiation, two new functionalities are introduced: Pyridine N-oxide and Pyridone. Among the three different tautomeric forms of pyridone it is emphasized the net charge separation in zwitterion pyridone-III as well as in pyridine N-oxide, thus resulting in a more p-doped.

To enhance the performances of graphene employed as a transparent conductor, a uniquely accessible route is to reduce the sheet resistance by operating a chemical doping. Therefore, it is essential to fingerprint the structure of the graphene layer and to understand the possible chemical modification for achieving high efficiency and stability in the charge transport process. For the nitrogen doped graphene (G:N), since the presence of C-N hetero-bonds into the aromatic platform, the main question is: *are there possibilities to functionalize the aryl carbon in such a way as to generate new electron-withdrawing groups, thus improving p-doping?* The outcomes arising from the present study confirm positively the hypothesis we set out. Figure 1 is a schematic representation of a crystalline grain, i.e. the graphene flakes, of the nitrogen-graphene (G:N) sheet as prepared and after aerobic photo-oxidation with singlet oxygen generated by Xe-lamp irradiation. In the schemes, in addition to the typical structural defects (carbon vacancy and Stone–Wales defects) and oxygen chemical functionalities (hydroxyl, carbonyl, carboxyl), the highlights are on the nitrogen functionalities. In as prepared G:N the N-functionalities are mainly graphitic (quaternary), pyridinic and pyrrolic; whereas light irradiation treatment in ambient conditions yields a more p^+^-doped graphene with some pyridinic groups converted to pyridine N-oxy and pyridone groups.

Looking ahead, from the side of the growth and the doping chemistries, both Raman and XPS data confirm (provided evidence) that graphene (G:N) incorporates N atoms during the growth and contains the “new” N-functionalities without introducing significantly C-sp^3^ defects in the C-sp^2^ basal-plane. Here, it is important to underline that Pyridinic-N and Pyrrolic-N occur mainly at the boundary of C-vacancy sites or, even more, at the edge of the graphene grains^19^. Therefore, since the Pyridinic-N is responsible for the activation of reactive sites leading to p^+^-doping, a higher defects density (C-vacancies and small grains) in G:N introduced during the growth allows for higher p-doping^33^.

**Figure 2:**
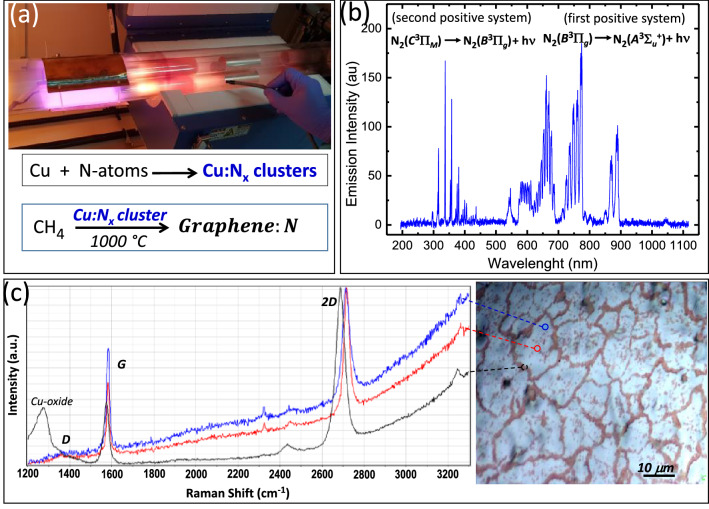
Growth of nitrogen doped graphene (G:N). (**a**) Image of the discharge plasma glow in nitrogen for copper nitridation and schematic of the graphene:N growth process using the Cu:Nx cluster (nitrogen solid source) in-situ prepared before growth. (**b**) Optical emission spectroscopy of the active species in the downstream of the N_2_ plasma. The emission intensity of first positive system (600–900 nm) is a measure of the N-atoms. (**c**) Raman spectra and optical microscopy of G:N on copper foil. The microscopy image shows the edge of the graphene grain as decorated (the dark line) by the copper oxide after long time exposure of the graphene covered copper foil to wet-air. The Raman spectra are collected from three different positions of the single grain: at the grain boundary (the black line), thus evidencing the copper oxide and within the grain for the wide single layer region (the blue line) and for the small bilayer island at the centre of the grain (the red line).

Figure 2a shows a photograph of the CVD reactor while performing the nitrogen plasma for the copper foil nitridation. Figure 2b shows an optical emission spectrum of the nitrogen plasma glow. The emission intensity of the first positive system is used for the evaluation of the nitrogen atoms amount in the plasma downstream where the copper foils are positioned^34,35^.

Optical microscopy and Scanning Raman spectroscopy were used for quality mapping the graphene layer (Fig. 2c). The microscope image shows the surface of the deposited graphene on copper foil after oxidizing by long time exposure to wet-air. The oxidation of the copper foil in correspondence of the graphene grain boundary decorates and draws the polycrystalline morphology of the graphene, whereas the isolated dark dots that spread throughout the graphene surface are local defective graphene spots, eg. carbon vacancies^13^. The size of graphene grains is around 10 µm. Two typical spectra with G (∼1582 cm^-1^) and 2D (∼2718 cm^-1^) peaks are observed without apparent D (∼1350 cm^-1^) peaks indicating the high quality of the graphene layer. The ratio I(2D)/I(G) = 1.8 and the G and 2D peak bandwidths of, respectively, 14 cm^-1^ and 27 cm^-1^ are fingerprint of a single layer graphene (blue line). The Raman spectrum with I(2D)/I(G) ∼1 (red line), recorded at the grain centre, is representative of bilayer. Most of the area is covered with single layer graphene, whereas, the presence of the small double layer island in centre of each grain confirms the under-layer nucleation mechanism described by Nie et al.^36^ The distribution density of double layer islands on graphene can be evidenced by enhanced optical contrast of the microscope image of graphene transferred on 300 nm SiO_2_/Si reported in Figure S1 together with the relative Raman analysis.

**Figure 3:**
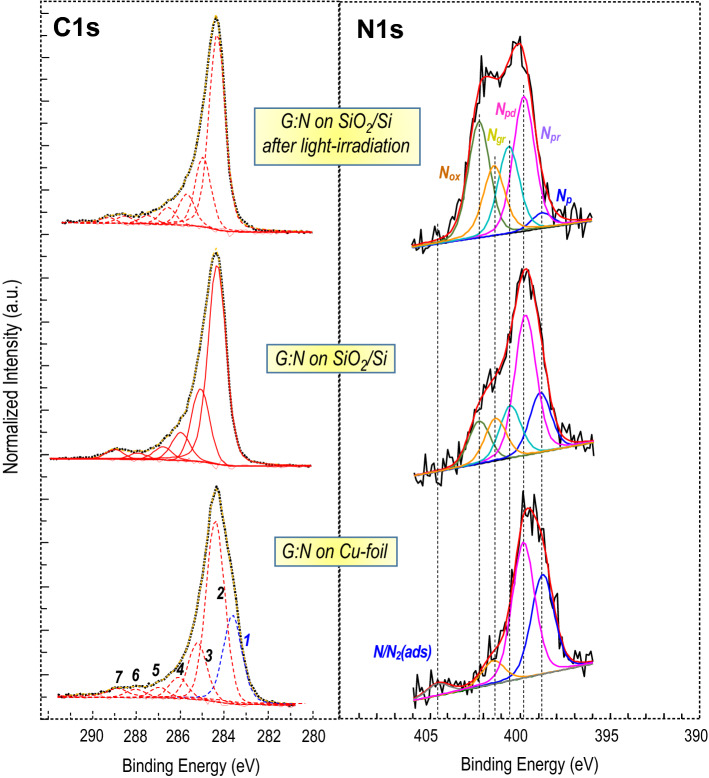
High-resolution (0.9 eV) C1s and N1s XPS spectra for single layer graphene as grown on Cu-foil, and transferred on SiO_2_/Si substrate before and after irradiation with Xe-lamp. **C1s**-spectra deconvolution of G:N on Cu foil is performed with seven peaks at 283.6 (1), 284.4 (2), 285.2 (3), 286.0 (4) 286.9 (5), 287.9 (6), 288.8 eV (7) that can be correlated to carbon atoms in the following configurations: C segregated in the Cu foil, C sp^2^, C–OH, C = N, C–O–C, C = O and O = C–O. However, after transferring on SiO_2_/Si and light irradiation, besides the disappearance of the component (1), peaks deconvolution slightly change because of introducing new chemical configurations. **N1s**-spectra deconvolution with corresponding typical configurations of N-containing functional groups, wherein pyridinic (*N*_*p*_) at 398.8 eV, pyrrolic (*N*_*pr*_) at 399.8 eV and graphitic (*N*_*gr*_) nitrogen at 401.5 eV are present in as grown graphene:N on copper foil. After transfer and light irradiation new peaks are added as needed to fit the observed large variation at the expense of pyridine nitrogen; specifically, at 400.7 eV and 402.3 eV assigned to Pyridone (*N*_*pd*_) and Pyridine N-oxide (*N*_*ox*_).

Figure 3 reports the XPS C1s and N1s acquired on single layer graphene as grown on copper and as transferred on Corning glass before and after Xe-lamp light irradiation. The quantitative estimation of the nitrogen content is about 1.5 at % the amount of carbon. Therefore, the labelling of the components used in the C1s-peak deconvolution resembles that of the pure pristine graphene (i.e. without nitrogen)^13^. Besides the small contribute of carbon solubilized in copper foil (peak 1 at 283.6 eV)^37^, there are no significant changes in the deconvolution going through the three samples. The assignment of the peak deconvolution indicates that the largest proportion of C-functionalities are always associated with presence of sp^2^ carbon with C = C bonds (284.4 eV) and carbon–oxygen as follows: C–OH (285.2 eV), C–O–C (286.9 eV), C = O (287.9), O = C–O (288.8 eV). The presence of carbon-to-nitrogen bonds adds a new peak at 286.0 eV ascribed to N = C bonds that are located in the π-conjugated graphene structure^37–39^. On the other side, significant changes are observed on N1s peak deconvolution.

Specifically, in the pristine graphene:N (G:N) grown on Cu substrate, besides the contribute at high binding energy (404.6 eV) due to adsorbed nitrogen^40,41^, it is recognized the presence of the three typical components of pyridinic (*N*_*p*_, 398.8 eV), pyrrolic (*N*_*pr*_, 399.8 eV) and graphitic/quaternary (*N*_*gr*_, 401.5 eV) nitrogen bonds^19,40^. The peak of graphitic nitrogen (*N*_*gr*_) is much lower than pyridinic (*N*_*p*_ ) and pyrrolic (*N*_*pr*_) nitrogen, thus the n-type doping with two p-electrons at the graphene π-cloud is low. Almost equivalent peak intensities are observed for N_p_ and N_pr_ signals for as grown graphene on copper foil. Following the graphene:N single layer transfer on Si/SiO_2_ substrate and the subsequent Xe-lamp irradiation, the N1s peak deconvolution needs two additional contributions at 400.7 eV and 402.3 eV assigned to Pyridone (*N*_*pd*_) and Pyridine N-oxide (*N*_*ox*_), respectively^19,38^. The appearance of these new N-functionalities takes place at the expense of pyridine nitrogen as evidenced by the strong reduction of the N_p_ energy peak, while N_pr_ peak, and, thus, the amount of pyrrolic nitrogen structures remains unchanged.

The observed evolution of the N1s peaks can be read in the chemical processes involving the interaction of G:N with singlet oxygen. In fact, the formation of singlet oxygen occurs on pristine G:N during both (a) the solubilisation of cupper foil by ammonium peroxydisulfate and (b) the light irradiation by Xe-lamp. As for the generation of single oxygen by interaction of G:N layer (still on TRT) with ammonium peroxydisulfate during the phase of copper solubilisation, the chemistry has been described in detail in previous studies^28,31,42^. The proposed chemistry is outlined in the following overall reactions (Eqs. (1) and (2)):

1 $$  {2}S_{2} O_{8}^{2 - } + 2H_{2} O \to ^{graphene:N} 4SO_{4}^{2 - } + 4H^{ + } + O_{2}^{ - } $$

2 $$  2 O_{2}^{ - } + 2H_{2} O \to ^{graphene:N} {}_{{}}^{1} O_{2} \left( {{}_{{}}^{1} \Delta_{g} } \right) + H_{2} O_{2} + 2OH^{ - } $$

and is based in the recombination of superoxide radicals ($$ O_{2}^{ - }$$) for the formation of singlet oxygen (^1^O_2_). Here, graphene:N surface is the catalyst that activate the hydrolysis of ammonium persulfate for the formation of superoxide radicals. Specifically, mainly pyrrole nitrogen acts on the neighbouring carbon atoms by increasing the charge density, thus generating an adsorption and activation center to form singlet oxygen^42^.

The graphene:N shows its catalytic action also in the photogeneration of reactive oxygen species (ROS) and, in particular, of singlet oxygen by molecular oxygen. In a recent paper, Yao and coworkers^29^ have evaluated the role of graphene functionalization in determining the ROS generation. In particular, they underline that the generation of ^1^O_2_ is driven by graphene photoexcitation with energy larger than the excitation energy of O_2_-ground state (^3^Σ_g_^-^) to ^1^O_2_ (^1^Δ_g_), which is of approximately 0.97 eV. This photosensitization mechanism has been reported as the most common means of singlet oxygen generation^43^. It is detailed that the energy transfer to O_2_ (^3^Σ_g_^-^) from an excited state of a sensitizer, which is formed by the light absorption of light in a specific wavelength region, results in the formation of both excited states O_2_(^1^Σ_g_^**-**^) and ^1^O_2_ (^1^Δ_g_). And, followed by the very fast spin-allowed ^1^Σ_g_^**-**^ → ^1^Δ_g_ deactivation that results in the complete formation of ^1^O_2_ (^1^Δ_g_). Moreover, it has been demonstrated that the graphene doping by heteroatoms, e.g., nitrogen, increase the photocatalytic efficiency^30,44^. The photochemical production mechanism of singlet oxygen on graphene:N can be depicted as follows:3

Thus, the completion of the ^1^O_2_-treatment process is carried out also on substrate-transferred graphene:N (glass or SiO_2_/Si substrates) by irradiation with Xe lamp. The effectiveness of the photocatalytic aerobic oxidation via singlet oxygen has been exploited by many for the realization of selective oxidative processes towards organic and biological molecules^30^.

Furthermore, nitrogen-doped graphene is an active catalyst also in the oxygen reduction reaction (ORR), in which again the singlet oxygen can play an important role^45,46^. In particular, since the high lifetime of ^1^O_2_ in the gas phase, reducing singlet oxygen is expected to facilitate the first electron transfer in ORR. The general scheme for the complete reduction of O_2_/^1^O_2_ molecules to OH^-^ is as follows:

4 $$ {}_{{}}^{1} O_{2} \left( {{}_{{}}^{1} \Delta_{g} } \right) + 2H_{2} O + 4e^{ - } \mathop \leftrightarrow \limits^{graphene:N} 4OH^{ - } $$

**Figure 4:**
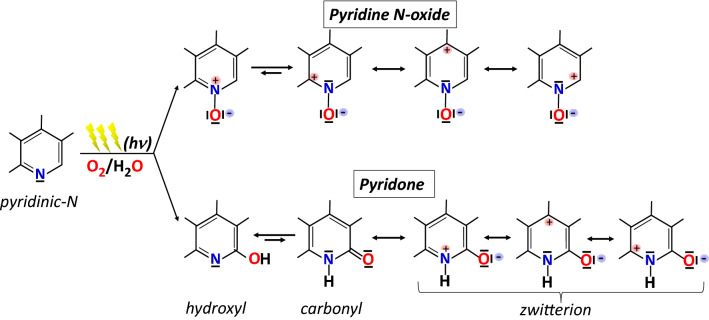
Simplified reaction scheme showing the formation of pyridine N-oxide and pyridone following the light irradiation of pyridinic nitrogen in the graphene network. The protonated tautomeric forms of pyridone, i.e., zwitterions, are involved together with pyridine N-oxide in the delocalization of positive charge throughout the whole π-conjugated system of graphene.

Nevertheless, the same oxidative processes can occur on the reactive sites present on the graphene surface; among these, the pyridine site is important as it can be oxidized simultaneously by aerobic photoirradiation to N-oxy pyridine and pyridine^27^, as schematized in Fig. 4.

The observed changes in nitrogen configurations, as assigned in Fig. 3, can be read in the simplified reaction scheme in Fig. 4, wherein the disappearance of the pyridine peak (*N*_*p*_ at 398.8 eV) is in favour of N-oxy pyridine (*N*_*ox*_ at 402.3 eV) and hydroxyl-pyridone (*N*_*pd*_ at 400.7 eV). While the other two pyridone-tautomers (carbonyl and zwitterion) with a protonated nitrogen, which resemble a quaternary nitrogen with a very similar XPS peak position to the graphitic nitrogen, contribute to the observed increased of the peak *N*_*gr*_ at 401.5 eV. The scheme emphasizes the role of the delocalization of the positive charge on the aromatic ring in both the oxidation to N-oxide pyridine and the formation of pyridone by the attachment of the OH group to the carbon atom in α-position to the pyridine nitrogen. The C1s XPS spectra shows that the C–OH, peak 3 at 285.5 eV, slightly increases after transferring on substrates. This confirms that carbon atoms close to pyridine nitrogen are the main active sites, among the different doping configurations of nitrogen, capable of further functionalizing graphene in the direction of p-doping^27^. Thus, the possibility of maximizing the introduction of N heteroatoms with pyridinic structure in the sp^2^ carbon lattice would maximize the p-doping effect.

**Figure 5:**
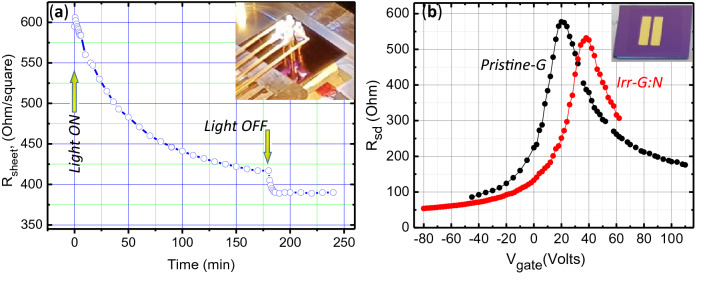
(**a**) Kinetics of sheet resistance change of G:N single layer on SiO_2_/Si substrate submitted to irradiation in air with a Xe-lamp. The sheet resistance stabilizes to its lowest value after about 3 h. The decrease, as soon as the lamp is turned off, is due to the thermal coefficient of the resistance*.* The inset shows the 4-probe Van der Pauw configuration. (**b**) Plot of the resistance between source and drain (R_sd_) as a function of gate voltage (V_g_) taken after thermal annealing (T = 200 °C) and in vacuum. The data in red and black are for irradiated graphene:N and pristine graphene, respectively. The inset shows the gold electrodes configuration of the FET device on SiO_2_/Si (channel length = 0.8 mm, width = 8 mm). The size of the single layer graphene:N is about 15 × 15 mm^2^.

The changes in G:N surface structure in the direction of a p-doping have been monitored by Raman spectroscopy and electrical characterization, both sheet resistance (Van der Pauw) and FET measurements. Figure 5 shows the sheet resistance variation, as measured in a 4-probe Van-der-Pauw configuration, during Xe-lamp irradiation exposure of a G:N single layer on Si/SiO_2_. The observed slow kinetics is consistent with complex formation pathway of singlet oxygen, whose concentration is determined not only from light irradiation (see Eq. 3) in the photocatalytic process but also from deactivation processes that includes both radiative decay and collisional decay, i.e., electronic-to vibrational transition by collision with N_2_, O_2_, H_2_O in air. The graphene:N surface reaction, i.e. the change of sheet resistance, is the result of the ^1^O_2_ adsorption kinetics on the pyridinic reactive site. In the same figure, the evidence of the p-doping following light irradiation is shown through the data from the field effect (FET) measurement, i.e. the shift of the Dirac point at high positive voltage (V_D_ = 39 Volts) for the light irradiated graphene:N when compared with pristine graphene (V_D_ = 20 Volts). The evaluation of hole carrier mobility has been done using the formula µ_h_ = (L/W)·C^-1^·V_D_^-1^·(dI_D_/dV_G_), where C = 120 µF·m^-2^ is the gate capacitance of the silicon dioxide dielectric layer (300 nm thick)^47–49^. The measured hole mobility of annealed pristine-G and light irradiated graphene:N are nearly equal and reach an average values of about 1350 cm^2^/(V·sec). Thus, the reduction of sheet resistance in irradiated graphene:N is mainly ascribed to the increase of hole carrier density.

**Figure 6:**
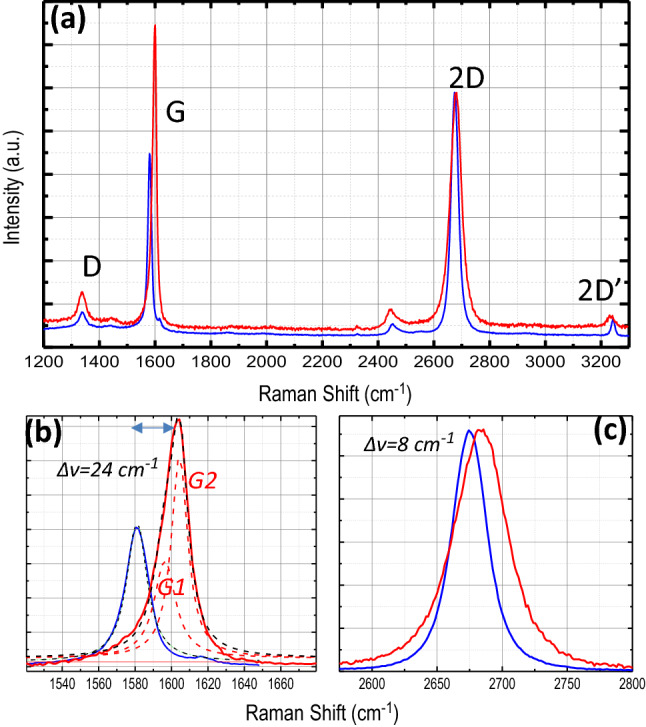
(**a**) Raman characterization of single layer graphene:N on Si/SiO_2_ before (blue line) and after (red line) irradiation by Xe-lamp (150 min). Spectra are normalized to the 2D peak. (**b**) Details of the G-peak Lorentzian-fit analysis. The asymmetric G peak of the sample after Xe-lamp irradiation and, consequently, the deconvolution in two peaks G1 and G2 (dashed lines) indicate charge inhomogeneity within the laser probe. (**c**) Details of 2D peaks showing the perfect symmetry and low up-shift after Xe-lamp irradiation.

The increase in hole carrier density (p-doping) is confirmed by the Raman analysis of single layer graphene on FET device. Figure 6 shows the Raman spectra of the graphene:N single layer, before and after Xe-lamp irradiation. In these spectra, the following features can be labelled: (i) the strong decrease of the 2D/G intensity ratio (from 1.4 to 0.8); (ii) the shift of the G peak that moves of 24 cm^-1^, from 1580 cm^-1^ to 1604 cm^-1^; (iii) the G peak asymmetry and Lorentzian-deconvolution in two narrow peaks (G1 at 1596 cm^-1^ and G2 at 1604 cm^-1^) after Xe-lamp irradiation; and (iv) the high-energy displacement of the 2D peak that moves of 9 cm^-1^, from 2675 cm^-1^ to 2684 cm^-1^. Similar results are observed from the Raman analysis of graphene:N transferred on glass before and after Xe lamp irradiation (Figure S2).

The simultaneous occurrence of these four events can be read in the more general picture of a strong increase in p-type doping (increase in the carrier density of holes) as described in the Raman "master paper" on the doping effect by Das et al.^48^. The observed asymmetry of the G-peak indicates the existence of charge inhomogeneity within the laser probe, i.e., on a scale of 1 µm^50^.

**Figure 7:**
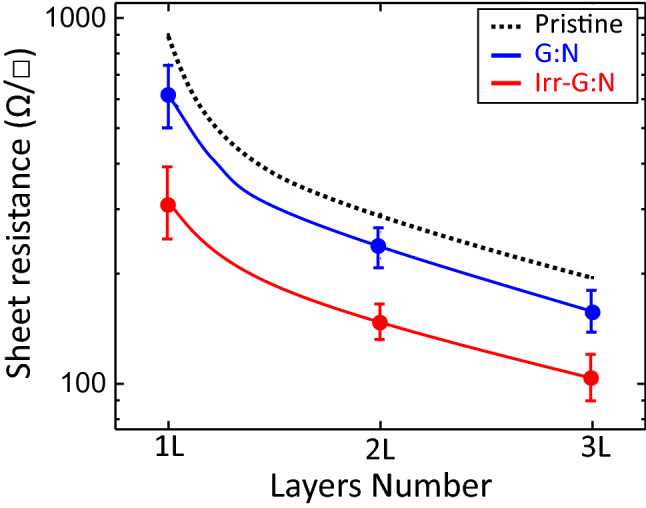
Sheet resistance data for nitrogen doped graphene multilayer before (G:N) and after Xe lamp irradiation (Irr-G:N). Dotted line (from Ref ^13^) is to guide eyes to read sheet resistance of pristine graphene without nitrogen doping.

According to ref.^49^, from these important Raman parameters we can estimate an hole doping density of about 1.3 × 10^13^ cm^-2^ for the irradiated graphene:N sample. This value is consistent with the average value of sheet resistance measured for a single layer of G:N after irradiation. In fact, by applying the simple Drude model of electrical conductivity, Rs (Ω/□) = 1/(n·µ·e), where n = 1.3 × 10^13^ (cm^-2^), µ = 1350 (cm^2^V^-1^ s^-1^) and e = 1.6 × 10^–19^ (A·sec), we extract the sheet resistance of 350 (Ω/□) that matches the measured values shown in Fig. 7.

Multilayer graphene samples on Corning glass substrates have been prepared by using the layer-by-layer procedure described in ref.^13^. Figure 7 reports the variation of the sheet resistance going from single-layer to three-layer graphene:N (blue dots) without and (red dots) with layer-by-layer light irradiation (each layer is irradiated after transfer). Multiple graphene layers act as parallel resistors, thus, providing a reduction of measured Rs values with the increase of the layer number. It is important to observe that the sheet resistance of the graphene:N layers, as transferred on Corning glass, already presents values lower than the sheet resistance of pristine multilayer graphene samples without nitrogen doping (dashed black line). The data show that the light irradiated graphene:N layers can provide a significantly low sheet resistance values due to the reaction mechanisms described in Fig. 4. Interestingly, the measured sheet resistance reduction is comparable to that obtained by other chemical doping procedures on multilayer graphene^13^.

## Conclusions

In summary, using a Nitrogen-doped graphene deposited from a direct CVD method, an efficient p-doping effect has been demonstrated through the selective photochemical activation of pyridinic nitrogen to N-oxy pyridine and pyridone. Notably, the sheet resistance of light-irradiated Nitrogen-doped graphene significantly reduces and can even reach a value as low as 100 Ω/□ for a three-layer graphene.

Unlike conventional chemical doping, where the graphene layer is submitted to chemical reaction with strong oxidants (HNO_3_, SOCl_2_,…) for the covalent doping or it is required to host molecules or particles for the non-covalent doping, the photochemical doping of graphene:N has the advantage to be easily applicable to any combined graphene-semiconductor device architecture. In addition to the capability of doping graphene on any surface device, this light-driven doping strategy has great potential to realize selective-area doping by photolithography mask or laser patterning.

## Material and Methods

### Graphene:N CVD growth, transfer and photoirradiation process.

The growth of nitrogen-doped graphene occurs in two phases: the plasma nitridation of copper foil and the catalytic CVD-growth. Both processes were carried out in the same quartz tube (i.d.10 cm) equipped with external capacitive-coupled electrodes for the plasma nitridation process and furnace for the CVD growth. The copper foil (25 µm thick, 10x10 cm^2^ size) was inserted in a quartz tube of the thermal-furnace CVD reactor. The quartz-tube was evacuated to a vacuum better than 10^-3^ torr and heated to 100 °C under an N_2_ gas flow of 200 sccm (0.2 torr) that was maintained for 20 min after the temperature was stabilized. The copper nitridation process was carried out in the nitrogen plasma downstream under the following conditions: N_2_ flow rate 200 sccm, pressure 0.2 torr, rf (13.56 MHz) power 250 watts, exposure time 30 min. Subsequently, the temperature was raised to 990 °C under an H_2_ gas flow of 10 sccm (0.05 torr) that was maintained for 20 min after the temperature was stabilized. The annealing process was followed by the graphene growth: CH_4_ (5 sccm) was added to the gas feed for a growth time of 20 min. After the growth phase, the furnace was moved from the growth-zone to realize the rapid cooling of the graphene/copper foil.

The graphene layer was then transferred to the substrate (Corning-glass, SiO_2_/Si) using the thermal release tape (TR-tape) procedure. The tape was placed on top of the graphene/copper foil and pressed by the laminator. The copper was etched away in an ammonium peroxydisulfate, ((NH_4_)_2_S_2_O_8_) solution (20 gr/liter) and the floating sheet of graphene/TR-tape was rinsed in DI water and air-dried. Hot-pressing (at ≈100°C) performed the graphene transfer onto substrates, thus the tape was then peeled off. Multilayer graphene samples on Corning glass for sheet resistance measurements were fabricated by the layer-by-layer transfer procedure described in ref.^13^.

The photoirradiation of the samples was performed under ambient laboratory conditions, in air using a 75 watt Xe-lamp (Oriel) with maximum wavelength emission at 470 nm. During light irradiation, the sheet resistance was monitored in real time to ensure the completion of the variation process.

### Characterizations

Raman spectra of the pristine graphene:N layers and of the photochemical modified layers were recorded with a LabRam HR (Horiba JY) system. Spectra on glass and SiO2/Si were measured at room temperature with 532 nm laser light and captured using 100x objective lens magnification with a focusing laser spot less than 1µm in diameter. Spectra of graphene on copper foil were collected using 473 nm laser**.** The laser power was kept at 1.0 mW to avoid laser-induced heating effect.

The surface chemical composition of doped samples was investigated by X-ray Photoelectron Spectroscopy (XPS), using a Theta Probe spectrometer (Thermo VG Scientific) equipped with a monochromatic Al Kα X-ray source (1486.6 eV) operated at 15 kV and a spot size of 400 µm, corresponding to a power of 70 W. Survey (0–1200 eV) and high-resolution (C1s, N1s) spectra were recorded in FAT (fixed analyzer transmission) mode at pass energy of 150 and 50 eV, respectively. All spectra were acquired at a take-off angle of 37° with respect to the sample surface. A flood gun was used to balance the surface charging. The C1s signal for the sp^2^ graphitic component of the C1s spectrum (284.4 eV) was used as internal standard for charging correction. The high-resolution spectra were fitted with mixed Gaussian-Lorentzian peaks after a Shirley background subtraction. The standard deviation in the peak position was ± 0.1 eV.

A Keithley 2400 Source Meter was used to monitor the electrical properties of graphene layers. Sheet resistance was measured in the 4-probe Van der Pauw configuration.

Single layer graphene on 300 nm SiO_2_ / Si substrates (*p*^+^*-*doping, resistivity ∼0.002÷0.005 Ohm·cm) were used for the field effect measurements. The FET device was fabricated by gold evaporation of the source and drain contacts (channel length 0.8 mm, width 8mm). Measurements were performed in air and in vacuum (better than 10^-3^ torr) with a Keithley 2400 analyzer at room temperature. A back-gate bias (*V*g) ranging from −80 to +110 Volts was applied on the Si side of the SiO_2_/Si substrate. Before measurement, the FET device was kept under vacuum overnight and treated by thermal annealing @ 200 °C. This allows to desorb surface species on graphene.

## Supplementary Information

Supplementary Information.

## Data Availability

Correspondence and requests for materials should be addressed to G.V.B.
