# Supplementary material for: Effective hole conductivity in nitrogen-doped CVD-graphene by singlet oxygen treatment under photoactivation conditions
Source: Sci Rep. 2022 May 24;12:8703. doi: 10.1038/s41598-022-12696-2 (PMC9130222; doi:10.1038/s41598-022-12696-2)
Supplement: Supplementary file 1 — Supplementary Information. [file 41598_2022_12696_MOESM1_ESM.docx]

**SUPPORTING INFORMATION**

**Effective hole conductivity in nitrogen-doped CVD-graphene by singlet oxygen treatment under photoactivation conditions**.

Giuseppe Valerio Bianco ^a,*^, Alberto Sacchetti ^a^, Marco Grande ^a,c^, Antonella D’Orazio ^c^, Antonella Milella ^a,b^, Giovanni Bruno ^a,^.

*^a^ Institute of Nanotechnology, CNR‑NANOTEC, Dipartimento di Chimica, Università di Bari, via Orabona, 4,*

*70126 Bari, Italy*

*^b^ Dipartimento di Chimica, Università di Bari, via Orabona, 4, 70126 Bari, Italy*

*^c^ Dipartimento di Ingegneria Elettrica e dell’Informazione, Politecnico di Bari; via Orabona,4 - 70123 Bari, Italy*

**Corresponding author:** Tel: +39-0805442082; E-mail: giuseppevalerio.bianco@cnr.it..

**Keywords:** Graphene; Nitrogen-Doped Graphene; Transparent Conductive Layer.

**Figure S1.** Optical microscopy and Raman analysis of N-doped graphene transferred on 300nm SiO_2_/Si substrate. Optical images of graphene on SI/SIO_2_ is representative of a typical CVD graphene foil with most of the area covered with single layer graphene (bright area), whereas, dark dots are small double-layer stacked islands grown according to the under-layer nucleation mechanism described by Nie et al. [1] . Figure (a) reports the Raman spectra measured at two different positions as shown in the optical microscopy image: at the single-layer area (blue line) and the centre of the small double-layer islands (red line). The relative peak heights (i.e. the ratio I_2D_/I_G_, of 2D band at ~2680 cm^-1^ and G band at ~1580 cm^-1^), that is a measure of the number of layers, changes from ~ 1.4 to ~ 1, going from single-layer graphene to the double-layer islands. Moreover, figure (b) shows that, in the case of monolayer (blue line), the G and 2D bands are well fitted by a single Lorentzian. Conversely, for double-layer graphene (red line) fitting of 2D band needs at least two Lorentzian peaks.

**Figure S2.** (a) Raman characterization of single layer graphene:N on Corning-glass before (blue line) and after (red line) irradiation by Xe-lamp (150 min). (b; c) Details of the G and 2D peaks, respectively, showing their shift (Δν)

**References**

[1] S. Nie, W. Wu, S. Xing, Q. Yu, J. Bao, S. Pei, and K. F. McCarty, Growth from below: bilayer graphene on copper by chemical vapor deposition, New J. Phys., 14, 093028; DOI:10.1088/1367-2630/14/9/093028 (2012).
